# Different Bilingual Experiences Might Modulate Executive Tasks Advantages: Comparative Analysis between Monolinguals, Translators, and Interpreters

**DOI:** 10.3389/fpsyg.2017.01870

**Published:** 2017-11-10

**Authors:** Sébastien Henrard, Agnès Van Daele

**Affiliations:** Department of Occupational Psychology, University of Mons, Mons, Belgium

**Keywords:** bilingual advantage, executive control, simultaneous interpretation, dual-language context, work activity

## Abstract

Many studies have shown that being bilingual presents an advantage in executive control. However, it appears that knowing two (or more) languages is not enough to improve executive control. According to the adaptive control hypothesis (Green and Abutalebi, 2013), the interactional context in which bilinguals behave is a key factor that modulates cognitive advantage in executive control. Translation and simultaneous interpretation are performed in a dual-language context: professional bi- and multilinguals use two or more languages within the same context (at work). Simultaneous interpretation differs from translation though, because of its higher level of time pressure, which increases the cognitive demands on executive control. The main objective of the present study is to investigate the relationship between simultaneous interpretation and some aspects of executive control. To this end, we compare the performance of three groups (60 interpreters, 60 translators, and 60 monolinguals) in five computerized tasks designed to assess different executive processes as well as the speed of information processing. The results show that the interpreters perform better than the monolinguals in all tasks and better than the translators in all tasks except for the one designed to assess flexibility. The results also show that the age variable does not have the same effect on performance in tasks designed to assess updating, flexibility, and resistance of proactive inhibition in bilinguals (both interpreters and translators), or in tasks designed to assess the speed of information processing and inhibition of a prepotent response in interpreters only. In addition to the advantage that being bilingual presents in some aspects of executive control, the results suggest that interpreters have an additional advantage that may be explained by the characteristics of their work activity (especially heavy time pressure) and by how much experience they have in this activity (in terms of magnitude of the bilingual management demands and amount of experience in managing the cognitive demands of simultaneous interpretation).

## Introduction

### Bilingual Advantage in Executive Control

There is a considerable literature demonstrating that bilingualism is beneficial in domains extending beyond language like executive control (Diamond, 2010). “*Executive functions (also called executive control or cognitive control) refer to a family of top-down mental processes needed when you have to concentrate and pay attention, when going on automatic or relying on instinct or intuition would be ill-advised, insufficient, or impossible* (Diamond, 2013, p. 135)". According to Green’s (1998) model, executive control is called upon in such management to inhibit activation of the conflicting non-target language. The hypothesis is that the daily use of interference management develops the functions and processes involved, thereby creating a bilingual advantage. Various studies have suggested a bilingual advantage in tasks requiring significant resistance to distractor interference (e.g., Bialystok et al., 2004; Costa et al., 2008). A bilingual advantage has also been shown for cognitive flexibility (Bialystok and Viswanathan, 2009), language and task switching (Prior and Gollan, 2011), as well as for working memory (Wodniecka et al., 2010; Luo et al., 2013). Some authors have also suggested that bilingualism provides continual practice in attentional control and results in improved functioning in adults. Thus, from a developmental perspective, bilingualism might contribute to cognitive preservation, protecting older adults from natural cognitive decline with age (Bialystok et al., 2004, 2006, 2007).

In most of the studies cited above, a significant difference between bilingual and monolingual groups has been interpreted as a direct effect of bilingualism on the cognitive functions. However, as pointed out by Kroll and Bialystok (2013), bilingualism ought not to be considered a dichotomous variable, given its multiple uses in everyday life. Rather, bilingualism is a dynamic phenomenon composed of multiple dimensions, and there is substantial variability within the bilingual population in terms of linguistic, cognitive, and social aspects, as well as work experience and education, making it difficult to compare the findings of various studies (Valian, 2015).

The empirical findings regarding a bilingual advantage in executive control are inconsistent (Hilchey and Klein, 2011; Paap and Greenberg, 2013). This is sometimes taken as evidence that the bilingual advantage simply does not exist (de Bruin et al., 2014) and that findings reflect controversial associations between bilingualism and cognitive control. Recently, several studies examining the relationship between bilingualism and executive control have shown more qualified results. These studies show that knowing two languages alone is not enough to improve executive control but that some characteristics of the daily use of bilingualism might lead to improvement in executive control (Hilchey and Klein, 2011; Kousaie and Phillips, 2012; Paap and Greenberg, 2013; Macnamara and Conway, 2014; Hernandez and Kohnert, 2015; Verreyt et al., 2016).

### The Adaptive Control Hypothesis

Green and Abutalebi (2013) advanced that the different interactional contexts of bilinguals’ conversational exchanges place varying demands on language control, which in turn adaptively alter their cognitive control capacities. According to this hypothesis called “the adaptive control hypothesis,” the challenge involved in bilinguals’ linguistic practices plays a key role in triggering more adaptive cognitive control. The authors distinguish three different interactional contexts: (a) the dual-language context, in which bilinguals use two languages (L1 and L2) within the same context (e.g., at home and/or at work); (b) the single-language context, in which bilinguals speak only one language in one environment, and therefore rarely switch languages (e.g., L1 at home and L2 at work); and (c) the dense code-switching context, in which bilinguals routinely mix the linguistic elements (e.g., words) of two languages within a single utterance (i.e., intrasentential code-switching). The adaptive control hypothesis suggests that bilinguals’ interactional context is a factor which modulates cognitive advantage in executive control. Specifically, bilinguals’ dual-language context involves a more complex and taxing level of control processes of goal maintenance, conflict monitoring, and interference suppression, and therefore facilitates more adaptive cognitive control than either the single-language or dense code-switching context. In support of this hypothesis, Macnamara and Conway (2014) and Hartanto and Yang (2016) found that bilinguals’ cognitive control and working memory were positively influenced by the magnitude of bilingual management demands and the amount of experience in managing the bilingual demands.

As discussed above, frequency of use, language switching, interactional context (dual, single, or dense code-switching), and amount of experience in managing the bilingual demands are crucial variables in the development of executive control in bilinguals (Yudes et al., 2011; Green and Abutalebi, 2013; Macnamara and Conway, 2014; Verreyt et al., 2016). Therefore, it seems natural to assume that the advantages of bilingualism for executive processes are more prevalent in bilinguals who have used two languages in the same context on a daily basis (e.g., as part of their profession) for many years.

### Simultaneous Interpretation as a Dual-Language Context

Within the research perspective reflected above, we chose to investigate simultaneous interpretation, a specific work-related activity, in terms of the implementation of bilingualism. Simultaneous interpretation is highly demanding in terms of executive control, requiring a large number of cognitive functions and processes to be activated simultaneously under heavy time pressure (Christoffels et al., 2006; Köpke and Nespoulous, 2006). This activity requires to continuously receive new information while simultaneously understanding speech, storing it in memory, and producing a translation of an earlier portion of speech (Gerver, 1976; Lambert, 1992; Moser-Mercer, 2000). Indeed, the simultaneous interpreter must listen to and understand speech in one language, holding it in memory until it is re-encoded to be produced in another language. At the same time, the interpreter utters the translation of a portion of speech encoded earlier. The resulting high demands on executive control may be said to make interpreters “experts in executive control” (Yudes et al., 2011).

Although interpreters are known to have a very good working memory capacity (Christoffels et al., 2006; Signorelli et al., 2012), little is known about their performances regarding updating or more general processes essential to the achievement of simultaneous interpretation, such as speed of information processing (Morales et al., 2015). Yudes et al. (2011) reported on two components of executive control, namely flexibility and inhibition, among professional interpreters, bilinguals, and monolinguals. Their results showed better performance by the interpreters in tasks evaluating cognitive flexibility and no difference between the three groups in tasks evaluating inhibition. These results are consistent with those of Köpke and Nespoulous (2006), who reported no advantage for professional interpreters in a Stroop task requiring participants to avoid interference from conflicting information. More recently, Morales et al. (2015) compared interpreters to bilinguals inexperienced in simultaneous interpretation or translation, reporting better performance among the former in updating information.

Overall, these results suggest that simultaneous interpretation, which reflects a particularly intensive use of bilingualism, is linked to components of executive control. However, given the few studies conducted to date on this topic, their relatively small numbers of subjects and the heterogeneity of the material used to evaluate executive control, definitive conclusions cannot yet be drawn.

## Objective and Hypotheses

The main objective of the present study is to investigate the relationship between simultaneous interpretation and some aspects of executive control by comparing the performance of professional interpreters to that of another group of bilinguals, namely translators, and to that of a group of monolinguals.

The use of a translators group is based on several methodological reasons. The first is their level of language mastery, as both interpreters and translators are required to have equivalent mastery of their second language by the end of their training. The second reason involves the control required for the type of professional activity performed by interpreters and translators, respectively. In most studies, bilingual participants are selected because they have mastered more than one language, without considering the context in which their bilingualism is used.

However, as discussed above, it may not be the mastery but rather the practice of two languages that has an effect on executive control (Green and Abutalebi, 2013; Kroll and Bialystok, 2013). If we follow Green and Abutalebi’s (2013) hypothesis, the context in which interpreters and translators work is the same dual-language context. Therefore, we hypothesize that interpreters and translators may show better performance in executive processes than monolinguals will. This “bilingual advantage” is related to the relative cognitive demand on the use of bilingualism in a specific context (in this case, a dual-language context).

Nevertheless, in this dual-language context, the work activity of interpreters and translators differs in terms of the relative cognitive demand on executive processes used to carry out their work activity. Translation, broadly defined, involves reformulating a message expressed in one language, the source language, in another language, the target language. Different types of translation may be distinguished based on input modality (visual vs. auditory), output modality (written vs. oral), and the parameters associated with input and output (e.g., simultaneous or consecutive). These parameters defining the type of translation involve different uses of executive processes (Ibáñez et al., 2010). The major difference between translation and simultaneous interpretation is time pressure. In simultaneous interpretation, time pressure increases the interpreter’s level of cognitive demand and executive processes used for this activity, whereas the translator is not affected by the flow of the speaker in the source language, and therefore he or she does not have to process information under time pressure. This temporal aspect leads to a difference in the processing speed and the speed at which information received has to be updated (Gile, 2009). Many studies have shown that time pressure in an activity (working or otherwise) leads to the establishment of two types of strategies: acceleration of information processing and filtering of information, by which only subjectively important information is considered (Edland and Svenson, 1993; Maule et al., 2000). Under time pressure, interpreters cannot process all the information, and so deliberately ignore information they consider less relevant. Finally, working under time pressure places interpreters at a greater risk of language interference than translators (Gile, 2009).

We hypothesize that the above differences between simultaneous interpretation and translation activities will be reflected in the performance of the respective tasks. By a cumulative effect of the dual-language context and the characteristics of work activity (time pressure), we predict that interpreters will perform better than translators in executive processes specifically involved in interpreting such as information processing speed, updating of information, and different types of inhibition (inhibition of a prepotent response and inhibition of proactive interference). However, as switching between two or more languages within the same context is the basis of both translation and interpretation, we predict no performance difference regarding this aspect.

To explain the nature of the bilingual advantage, a more developmental perspective may be required. Indeed, with the amount of experience in a dual-language context, moderation of the effects of age variable on performance in tasks assessing executive control may be expected. As mentioned above, bilingualism is considered by various researchers to be a source of cognitive reserve, protecting the bilingual against cognitive decline (Bialystok et al., 2004, 2006, 2007). As previously suggested, simultaneous interpretation may have a cumulative effect with bilingualism. If so, we hypothesize that the magnitude of bilingual management demands and the amount of experience in managing the simultaneous interpretation cognitive demands (called below “accumulated experience” in simultaneous interpretation) moderate age variable effects on performance in tasks assessing executive processes specifically involved in that work activity.

However, to determine the relationship between accumulated experience and performance in tasks assessing executive control, it must be ascertained whether there is any difference in performance among interpreters, translators, and monolinguals early in their respective careers. The existence of such differences might suggest that interpreters, translators, and monolinguals are not characterized by the same cognitive abilities at that early stage, which would amount to a confounding variable, preventing conclusions about the link between accumulated experience and performance in interpreters.

## Materials and Methods

### Participants

A total of 180 participants, divided into three groups (60 interpreters, 60 translators, and 60 monolinguals), took part in this study. No participant had an uncorrected visual impairment or a disorder which might interfere with executive control. No participant reported any history of cardiovascular, psychiatric, or neurological disease. All participants were informed about the purpose of the study and gave free consent. The research was approved by the Ethics Committee of the University of Mons (Faculty of Psychology and Educational Sciences).

The 60 interpreters (23 men and 37 women) were all professionals from international institutions in Brussels or Luxembourg. They all had at least a 4-year higher education degree (university or other). They all had French as their mother tongue or first language learned. Their interpreting experience ranged from 1 to 43 years (*M* = 18.57 years; *SD* = 12.07). They ranged in age from 24 to 65 years (*M* = 44.28 years; *SD* = 11.68). Excluding French, we counted 14 languages (L2): Dutch, German, English, Spanish, Russian, Italian, Slovenian, Danish, Romanian, Polish, Norwegian, Swedish, Portuguese, and Greek.

The performance of the 60 interpreters was compared to that of two control groups. The first consisted of 60 bi- or multilingual translators (26 men and 34 women) from Belgium who all had at least a 4-year higher education degree (university or other). None had worked as an interpreter. They all had French as their mother tongue or first language learned. Their translation experience ranged from 2 to 41 years (*M* = 21.22 years; *SD* = 11.55). They were between the ages of 25 and 65 years (*M* = 44.98 years; *SD* = 11.83). French excluded, there were 8 languages (L2) in the sample: Dutch, German, English, Spanish, Russian, Italian, Chinese, and Danish. The additional languages were learned between birth and 14 years of age for interpreters and between birth and 18 years of age for translators. The two bilingual groups were considered equivalent in terms of bilingual proficiency, as both had passed similar evaluations in terms of mastery of a second and third language at the end of their training. While this does not guarantee the same level of performance throughout their careers, it does give an indication of their level of mastery. Every participant in the two bilingual groups used two or three languages every day at work.

The second control group consisted of 60 monolingual participants (29 men and 31 women) with university education and a range of professions (including lawyer, architect, historian, banker, educator, and psychologist) whose mother tongue was French. They were between the ages of 25 and 65 years (*M* = 44.02 years; *SD* = 11.58), and their work experience ranged from 1 to 43 years (*M* = 17.78 years; *SD* = 10.62). They lived in a multilingual country (Belgium) but had only a passive and minimal knowledge of a second language. They were considered monolingual as they had not mastered a second language and were unable to hold a conversation in a second language, although they had been exposed to a second language passively in the workplace or through the media.

The three groups were statistically comparable in terms of sex (χ^2^ = 1.22; *p* = 0.543), seniority [*F*(2,177) = 1.487, *p* = 0.229], educational level [*F*(2,177) = 0.512, *p* = 0.600], and age [*F*(2,177) = 0.109, *p* = 0.897]. The participant characteristics are summarized in **Table 1**.

**Table 1:** Mean, standard deviation, and *p*-value of ANOVA.

	INT (*N* = 60)	TRAN (*N* = 60)	MON (*N* = 60)	*p*-value
Men/women	27/33	26/34	29/31	0.543
Age	44.3 (11.7)	45 (11.8)	44 (11.6)	0.897
Professional experience	18.6 (12)	21.2 (11.6)	17.8 (10.6)	0.229
Education	16.6 (1.4)	16.5 (1.2)	16.7 (1.2)	0.600

*INT, Interpreters; TRAN, Translators; MON, Monolingual.*

### Stimuli and Procedure

Five computerized tasks were performed by each participant in random order. These tasks were selected to access different executive processes essential to simultaneous interpretation as well as executive processes not involved in translation, thus helping differentiate between the executive processes used in interpretation and translation. The tasks included a computerized version of the Brown–Peterson task, a task of simple reaction time and three tasks assessing some aspect of executive control: letter memory, antisaccade, and the plus–minus task of Miyake et al. (2000). These tasks were selected because, according to Miyake et al. (2000), they are highly correlated with the executive function they are supposed to evaluate. They therefore represent the best approximation of the executive functions we wanted to evaluate.

The tasks were created and presented by E-prime 2.0^®^ software. Each participant was tested individually during a single session lasting approximately 75 min.

#### Reaction Time Task

Participants’ reaction times were measured in two modalities: firstly, by pressing the space bar on the keyboard as soon as possible in response to a cross appearing in the center of the screen, and secondly, by producing the word cross in a voice key as soon as possible in response to the same cross appearing in the center of the screen. There were 50 trials for each modality. The time between each trial varies between 1,000 and 4,000 ms in steps of 250 ms. The score used for data analysis was the average response time across the two modalities. We regarded this task as accessing the speed of information processing.

#### Letter Memory Task

This task, an adaptation of Morris and Jone’s (1990), consisted in serial lists of letters with a presentation time of 2,000 ms per letter. The participant, not knowing in advance the length of the list, was asked to recall the last four letters in each list presented. The test comprised two parts: an initial control portion of four trials with four letters and a second part with 12 trials of varying numbers of letters (5, 7, 9, or 11 letters). The score used for data analysis was the percentage of letters correctly recalled. According to the model of Miyake et al. (2000), we regarded this task as accessing the updating of information.

#### Antisaccade Task

An adapted version of the antisaccade task of Roberts et al. (1994) was used. For each item, an attachment point was displayed on the center of the screen for a time ranging between 1,500 and 3,000 ms in steps of 250 ms. A visual cue (a black square) was presented on one side of the screen for 225 ms, followed by the presentation of the stimulus (an arrow inside an open square) on the opposite side of the screen for 150 ms. The participant was required to indicate the direction of the arrow on the keyboard. As the arrow appeared only 150 ms before being masked, the participant had to inhibit the reflex response [reflecting resistance to a prepotent response in Friedman and Miyake’s (2004) model] to look at the initial cue, as this would make it more difficult to identify the direction of the arrow. The cues and targets were both presented 15 cm away from the fixation point (on opposite sides) and the participants were seated 50 cm from the computer monitor (thus, the total subtended visual angle from cue to target was approximately 33.4°). This task consisted of 80 trials. The score used for data analysis was the percentage of correct answers. According to the model of Friedman and Miyake (2004), we regarded this task as accessing the inhibition of a prepotent response.

#### Plus–Minus Task

This task, adapted from Spector and Biederman’s (1976), included three lists of 30 numbers (10–99). For the first list, the participant had to add three to each number as quickly as possible. For the second list, the participant had to subtract three from each number as quickly as possible. For the third list, the participant had to alternate between adding three and subtracting three. This task assessed flexibility by comparing both performance and response time (reflecting shift cost) in the switching task (list 3) to those in the first two tasks (lists 1 and 2). The shift cost was calculated as the difference between the completion time of correct responses for the third list and the average completion time of correct responses for each of the first two lists. According to the model of Miyake et al. (2000), we regarded this task as accessing flexibility.

#### Brown–Peterson Task

In this 24-trial task, the participant had to memorize a series of three visually presented consonants, complete an interfering task (i.e., digit inversion), and then repeat the three consonants originally viewed. The difficulty of the task varied depending on the length of the interval between the presentation of the three consonants and their recall, the interval being 5, 10, or 20 s in length (Peterson and Peterson, 1959). The score used for data analysis was the percentage of correct consonants recalled. According to the model of Friedman and Miyake (2004), we regarded this task as accessing the resistance of proactive inhibition.

### Data Analysis

All data were processed using SPSS 21^®^ software. For each task where reaction times were used, data reduction was first performed following the recommendations of Ratcliff (1993) for dealing with outliers and errors in Reaction time tasks: (1) trials with incorrect responses were excluded from the Reaction time analyses (3.15% of trials); (2) Reaction times shorter than 100 ms or longer than 2,000 ms were removed from analyses (0.004% of trails with correct responses); (3) Reaction times more than two SDs below or above each participant’s mean for each experimental condition were discarded as outliers (0.014% of remaining trials).

Five indicators were used as a reflection of participants’ performance: (a) average reaction time in the Reaction times task; (b) percentage of letters correctly recalled in letter memory; (c) percentage of correct answers in the Antisaccade task; (d) average shift cost in the Plus–minus task; and (e) percentage of letters correctly recalled in the Brown–Peterson task. Thus, our experimental design had five dependent variables (DVs) and one independent variable (IV), namely group (interpreters vs. translators vs. monolinguals). All DVs inter-correlated significantly (with *r* ranging from -0.443 to 0.430, *p* < 0.01).

To examine the relationship between bilingualism, simultaneous interpreting, and executive processes, a multivariate analysis of variance (MANOVA) was performed by introducing the experimental group and the DVs into the model. The *post hoc* Bonferroni’s test was used to determine between which groups significant differences occurred.

To test the moderation of age variable effects^1^ on executive processes, we performed a multiple analysis of covariance (MANCOVA). We used the same experimental design but introduced age as a co-variable. The objective was to show that the effect of age variable on executive decline varied according to group.

Before performing the MANCOVA, we verified that age variable had an effect on performance. Age and performance were found to be significantly correlated (with *r* ranging from -0.332 to 0.528, *p* < 0.01). We then determined whether or not there were differences in performance among the younger participants in each group (25–34 years; *n* = 45; 15 participants from each group). A MANOVA was performed by introducing the experimental group and the DVs into the model. Using Wilks’ Lambda criterion, the DVs combined were not significantly affected by the experimental group [*F*(10,76) = 0.949, *p* = 0.494, η^2^ = 0.111], indicating that performance did not differ significantly among the younger participants of the three groups.

## Results

Using Wilks’ Lambda criterion, the DVs combined were significantly affected by the experimental group [*F*(10,346) = 8.714; *p* = 0.0001, η^2^ = 0.201], indicating a significantly different performance among the three groups of participants. Subsequent analyses of each DV separately showed results consistent with those obtained for the combined DVs. These analyses revealed significant differences among the groups for each task. *Post hoc* analyses conducted using the Bonferroni’s test showed that these significant differences in performance varied according to the task (**Table 2**).

**Table 2:** Means, standard deviation, MANOVA results (*F-* and *p*-values), and Bonferroni’s *post hoc* test results (*p*-value) for interpreters, translators, and monolinguals by performance indicator.

	Mean (*SD*)			*F*	*p*	η^2^	*Post hoc* test		
	INT	TRAN	MON				INT vs. TRAN	INT vs. MON	TRAN vs. MON
Reaction time (ms)	309 (24)	338 (32)	351 (48)	21.35	**0.0001^∗^**	0.194	** 0.0001^∗^**	**0.0001^∗^**	0.152
Plus–Minus (ms)	52 (80)	65 (47)	113 (70)	13.24	**0.0001^∗^**	0.130	0.874	**0.0001^∗^**	**0.001^∗^**
Letter memory (%)	92.92 (5.81)	86.18 (6.71)	83.1 (10.94)	22.84	**0.0001^∗^**	0.205	** 0.0001^∗^**	**0.0001^∗^**	0.119
Antisaccade (%)	85.38 (8.28)	79.83 (10.83)	78.38 (15.97)	5.57	**0.005^∗^**	0.059	**0.04^∗^**	**0.006^∗^**	1
Brown–Peterson (%)	96.67 (2.99)	93.36 (4.7)	89.93 (8.92)	18.47	**0.0001^∗^**	0.173	**0.01^∗^**	**0.0001^∗^**	**0.007^∗^**

*INT, Interpreters; TRAN, Translators; MON, Monolinguals. ^∗^*p* < 0.05.*

**FIGURE 1:**
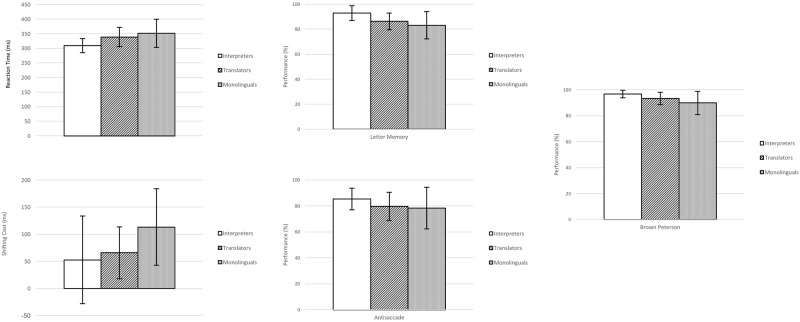
Means and standard deviation of interpreters, translators, and monolinguals in reaction times, shifting cost, letter memory, antisaccade and Brown Peterson.

Specifically, the data analysis showed that the interpreters performed significantly better than the monolinguals on all tasks, and significantly better than the translators on all tasks except the Plus–minus task. Finally, the translators performed significantly better than the monolinguals in the Plus–minus and Brown–Peterson tasks (**Figure 1**).

Using Wilks’ Lambda criterion, the DVs combined were significantly affected by the interaction between group and age variables [*F*(10,340) = 5.194; *p* = 0.0001; η^2^ = 0.133]. These analyses revealed that the effect of age variable on performance varied depending on group (**Table 3**).

**Table 3:** Results (*F*, *p*, η^2^) of MANCOVA analysis for each independent variable.

	Age × Group		
	*F*	*P*	η^2^
Reaction times	7.353	**0.001^∗^**	0.078
Antisaccade	5.882	**0.003^∗^**	0.063
Plus–minus	3.667	**0.028^∗^**	0.04
Letter memory	8.613	**0.0001^∗^**	0.09
Brown–Peterson	7.320	**0.001^∗^**	0.078


*^∗^*p* < 0.05.*

To determine where the differences in age variable effects among the three groups were located, pairwise comparisons were performed. With regard to the comparison between the interpreters and translators groups using Wilks’ Lambda criterion, the DVs combined were significantly affected by the interaction between group and age variables [*F*(5,112) = 4.03, *p* = 0.002; η^2^ = 0.152]. Comparing the interpreter and monolingual groups using Wilks’ Lambda, the DVs combined were also significantly affected by the interaction between group and age variables [*F*(5,112) = 10.49; *p* = 0.0001; η^2^ = 0.319]. Finally, the comparison between the translators and monolinguals groups using Wilks’ Lambda revealed that the DVs combined were significantly affected by the interaction between group and age variables [*F*(5,112) = 3.372; *p* = 0.003; η^2^ = 0.144].

**FIGURE 2:**
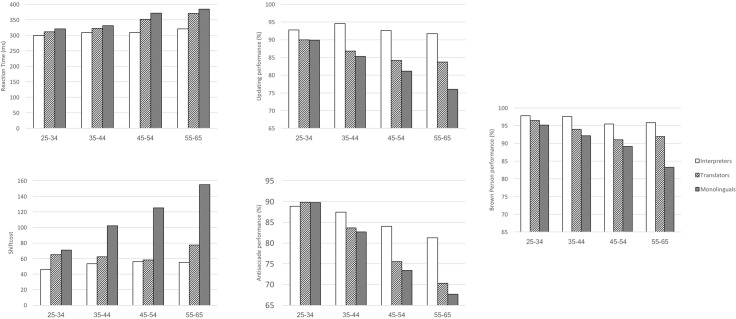
Means of interpreters, translators and monolinguals in reaction times, shifting cost, letter memory, antisaccade and Brown Peterson by age group.

These results showed that the effect of age variable on performance in the interpreters group differed regarding the Reaction times and Antisaccade tasks. The results also showed that age variable did affect in the same way the performance in the two bilingual groups (interpreters and translators) for the Letter memory, Plus–minus, and Brown–Peterson tasks (**Figure 2**). The results are summarized in **Table 4**.

**Table 4:** Results (*F*, *p*, η^2^) of pairwise MANCOVA analysis for each independent variable.

	Age × Group		
	*F*	*p*	η^2^
**Interpreters vs. Translators**			
Reaction times	14	**0.0001^∗^**	0.108
Antisaccade	9.525	**0.003^∗^**	0.076
Plus–minus	0.005	0.945	0.0001
Letter memory	3.276	0.073	0.027
Brown–Peterson	1.251	0.266	0.011
**Interpreters vs. Monolinguals**			
Reaction times	10.874	**0.001^∗^**	0.086
Antisaccade	9.259	**0.003^∗^**	0.074
Plus–minus	4.588	**0.034^∗^**	0.038
Letter memory	14.914	**0.0001^∗^**	0.114
Brown–Peterson	11.307	**0.001^∗^**	0.089
**Translators vs. Monolinguals**			
Reaction times	0.479	0.490	0.004
Antisaccade	0.703	0.404	0.006
Plus–minus	7.352	**0.008^∗^**	0.06
Letter memory	5.853	**0.017^∗^**	0.048
Brown–Peterson	6.127	**0.015^∗^**	0.05

*^∗^*p* < 0.05.*

## Discussion

### The Bilingual Advantage

If a bilingual cognitive advantage exists, it would translate into better performance in tasks assessing executive processes for all bilingual groups, as opposed to any monolingual group. Regarding the Brown–Peterson and Plus–minus tasks in the present study, the results show that the interpreters and translators were significantly more efficient than the monolinguals. These results suggest the existence of a relationship between the dual-language context of bilingualism and these activities.

Furthermore, shift costs seem, as expected, linked with bilingualism. We found no significant differences between interpreters and translators in this regard. Both bilingual groups performed significantly differently from monolinguals, suggesting a relationship between professional bilingualism and performance in the task. As shown by numerous studies (Bialystok and Viswanathan, 2009; Garbin et al., 2010; Prior and MacWhinney, 2010; Prior and Gollan, 2011), bilinguals exhibit lower shift cost than monolinguals do. Recently, Verreyt et al. (2016) showed that performance in tasks assessing executive control in bilinguals depended on the degree of switching between languages. They showed that bilinguals who switched more often between two languages performed better in tasks assessing the management of interference. More than the level of mastery of the languages, it may be the experience and the way languages are used which improves performance on tasks assessing executive control (Green and Abutalebi, 2013). Our results point in the same direction. The ability to disengage from one language to engage in another, whether as an interpreter or a translator, may lead to greater development of this specific executive function in bilinguals compared to monolinguals. Nevertheless, the present data should be regarded with a degree of caution. Indeed, in this task, the SD is greater than the mean score for interpreters who show a great variability of performance in this group. Some interpreters perform the switching task faster than the two simple tasks. Even if there seems to be a link between bilingualism and performance in the Plus–minus task, the interpreters’ group presents very heterogeneous results, so we should be cautious about our conclusions.

Regarding resistance to proactive interference, the results seem to show a double effect of bilingualism and work activity. Interpreters performed better than translators, who in turn performed better than monolinguals. Our results suggest that resistance to proactive interference (assessed by the Brown–Peterson task) is linked to bilingualism but also to interpreting experience. Few studies, to our knowledge, have explored a possible link between bilingualism and proactive interference, but Bialystok and Feng (2009) used a variant of the Brown–Peterson task to show that there was no difference in performance among monolingual and bilingual adults.

To explain these results, the task itself must be observed. Participants are required to remember three letters, complete an interfering task of counting, and then repeat the three original letters. We would like to point out that many interpreters have emphasized that, when asked, they can retain three letters with very little effort. Acronyms and initials are commonly used in the work of interpreters, and of some translators in market sectors. The demand not being the same for the two groups, this task may not measure the same thing in bilingual and monolingual groups. Consequently, the results obtained may be due to job-related knowledge that is a benefit of work experience, rather than bilingualism.

### The Interpreter Advantage

If interpreting experience modulates the bilingual advantage, we would expect better performances for interpreters than the other two groups in different tasks assessing executive processes specifically involved in simultaneous interpretation. Our results point in the same direction as those already reported by various researchers, suggesting that interpreters perform better than others in tasks assessing different aspects of executive control (Köpke and Nespoulous, 2006; Yudes et al., 2011; Morales et al., 2015). Regarding the Letter memory, Reaction times, and Antisaccade tasks, the present results showed that interpreters were significantly more efficient in these tasks than the two other groups, which performed similarly to each other. These results suggest the existence of an “interpreter advantage.”

Given the limited capacity of working memory, the interpreter must be able to constantly update the information received, i.e., the speech of the speaker. Morales et al. (2015) showed that interpreters performed better than bilingual non-interpreters in an N-Back task evaluating updating, a function specifically used in simultaneous interpretation. The results of the Letter memory task in this study confirm this interpreter advantage.

The time pressure related to the activity of simultaneous interpretation, and the need to constantly update the information received require that information be processed quickly. To our knowledge, no previous research has specifically studied the speed of information processing by interpreters. However, Christoffels et al.’s (2006) study showed that interpreters were faster than students and language teachers in a naming task. Our results for simple reaction time showed that the interpreters processed information faster than the translators and the monolinguals. This result can be linked to the temporal characteristics of simultaneous interpretation. Indeed, the production of the target message often begins a few seconds after the uttering of the source message. This time lag, most frequently called ear-voice span (EVS), is measured by the number of words or seconds between incoming information (input) and outgoing information (output). For interpreters, the average EVS has been shown to vary between 4 and 5.7 words (Goldman-Eisler, 1972; Gerver, 1976); in terms of time, the EVS reflects a 2- to 3-s delay (Christoffels and De Groot, 2004). Therefore, to achieve good quality simultaneous interpretation, it is mandatory to process information quickly, as seems to be reflected in the performance of the tasks assessing processing speed.

Regarding the Antisaccade task, our results seem to show that the resistance to a prepotent response is more efficient in interpreters than in the two other groups. In fact, interpreters and translators do not use the same inhibitory processes in their work activity. Under time pressure, interpreters cannot process all the information, and so deliberately ignore information they consider less relevant for understanding speech. The resistance to a prepotent response likely reflects a process for simultaneous interpretation which is not as active in translation. Supporting this argument, the lack of difference between monolinguals and bilinguals was highlighted by Paap and Greenberg (2013) or Paap et al. (2015), who compared monolinguals and bilinguals in an antisaccade task and did not find any differences between the groups. Thus, it seems that the results are related to the activity of simultaneous interpretation and not to the effects of the dual-language context. Resistance to a prepotent response as voluntary inhibition is not the basis of a conventional process of bilinguals, or even of translators, but the prerogative of interpreters.

To confirm this proposal, a more detailed analysis of the cognitive processes involved in simultaneous interpretation is required. At present, it seems that the simultaneous interpretation activity can affect performance in tasks assessing different executive processes.

### The Effects of Accumulated Experience

We observed no significant difference in performance in tasks assessing executive processes among our younger participants (aged 25–34 years) who were in the early stages of their careers. As Kroll and Bialystok (2013) point out, at this age, young adults are at their “peak performance” in different tasks assessing executive process (see also Bialystok et al., 2005, 2008; Morton and Harper, 2007; Colzato et al., 2008; Hilchey and Klein, 2011). Another explanation recently offered by Valian (2015) is that younger participants are themselves in a phase of accumulation of rich cognitive experiences. These new experiences require planning, inhibition, and flexibility, and developing executive processes may mask the bilingual advantage. Nevertheless, analyzing by age, we can notice that significant differences appear after 35 years of age, but the interpreters tend to be better before that. This means that a difference must be considered originally, even if this difference widens between groups over time.

The significant differences between the groups appeared later in the present data and may be explained (at least partially) by the moderation of the age variable effect on performance in Letter memory, Plus–minus, and Brown–Peterson tasks in the bilingual groups (translators and interpreters), and in Reaction times and Antisaccade tasks in the interpreter group. The effects of age variable on performance were not the same for the letter memory, plus–minus, and Brown–Peterson tasks among the bilinguals. As expected, these results reflect the requirement of the dual-language context as the basis for developing cognitive reserve as protection against executive decline.

In addition, we found that age variable did not have the same effect on performance in Reaction times and Antisaccade tasks among interpreters. This result suggests that the accumulated experience in simultaneous interpretation may be related to a lower decline in executive processes involved in this activity (i.e., speed of information processing and inhibition of a prepotent response).

In a recent review of the literature, García (2014) developed the idea of the interpreter advantage hypothesis. Our study supports this hypothesis by showing that the interaction between the context of use (in this case, a dual-language context), the characteristics of the work activity (mainly heavy time pressure), and accumulated experience in this activity seem to form the basis of the relationship with executive control. As we have seen, time pressure demands greater processing speed and filtering of the information processed (Edland and Svenson, 1993; Maule et al., 2000). The accumulated experience in using these two processes through the simultaneous interpretation activity appears to moderate the effects of age variable on these processes.

These results can also be linked to a series of results in neuroimaging studies showing that, in interpretation students training in a 15-month program, there is an increase in gray matter volume in brain regions known to be involved not only in semantic processing but also in learning, motor control, and in a variety of domain-general executive functions (Hervais-Adelman et al., 2011, 2015a). The recruitment of similar circuits during language and executive control provides powerful evidence that the continuous demand of language control in the multilingual brain and associated experience-dependent plasticity could underlie the non-linguistic executive advantages that have been observed in bilingual individuals, advantages that may also be protective in defying challenges posed by aging and even disease (Hervais-Adelman et al., 2015b). Future studies investigating interpreters could shed light on the relationship between performance in task evaluating executive control and modification of brain regions involved in this activity.

## Conclusion

In this research, we investigated the relationship between simultaneous interpretation and executive control. The main results confirm some of those already reported in the literature. Simultaneous interpretation appears to be positively linked to executive control. In addition, the results seem to confirm that, beyond a bilingual advantage related in our study mainly to the importance of cognitive demand on the use of bilingualism in a specific context (a dual-language context), there is an interpreter advantage which is also related to the effects of the work activity (especially heavy time pressure) and the accumulated experience (magnitude of bilingual management and amount of experience in managing bilingual cognitive demands) in this activity (in terms of magnitude of bilingual management demands and amount of experience in managing the simultaneous interpretation cognitive demands).

Like Yudes et al. (2011), we have shown that the interpreter advantage is not general, but restricted to the precise cognitive operations needed to perform the interpreting task, and may be bound to the heavy time pressure of the activity.

The present results should be regarded with a degree of caution, as certain limitations to the research are worth noting. Firstly, this research was based on a transverse approach focusing on a large number of participants and strict control of experimental groups. While we took the precaution of checking for a difference in performance between early career groups, this does not preclude a possible selection bias. In fact, it appears that even if interpreters aged from 25 to 34 years already tend to have better performances than the two other groups, this difference was not significant. Moreover, we did not control other cognitive processes that may have influenced groups’ performance.

Secondly, the choice of tasks included in the present research was justified by reference to the models of Miyake et al. (2000) and Friedman and Miyake (2004). We selected tasks for which performance may be explained in terms of the contribution of separate executive functions or processes (flexibility, updating, speed of information processing, inhibition of a prepotent response, and resistance to proactive inhibition). This choice of tasks nevertheless raises certain questions, and we are aware of the possible influence of non-executive processes on the performance of participants in the selected tasks. This seems especially plausible for the Brown–Peterson task since we observed that interpreters and translators relied on acquired knowledge. Indeed, the three letters to be remembered often referred to business or were organizational acronyms encountered in the work context, facilitating their retention. Furthermore, it should be noted that the tasks are mainly verbal (only the Antisaccade task is not), which may have led to interferences between verbal abilities and executive functioning (Bialystok et al., 2004). To avoid this, further research would have to use only non-verbal tasks or independent procurement tasks specifically targeting non-executive processes.

In conclusion, the present results do not question the bilingual advantage but highlight the distinction between the fact of being bilingual and the way in which bilingualism is used. In a first approach intending to clarify the context in which bilingualism is used, Green and Abutalebi (2013) distinguish among the different executive processes used in a dual-language context, single-language context, and dense code-switching context. Our study provides additional insight by showing that even in a dual-language context, a bilingual advantage in various executive processes depends partly on the activity carried out.

More broadly, further research is needed to determine the mechanisms underlying an interpreter advantage in tasks assessing executive control and to identify whether and which other functions or cognitive processes may be modulated by the activity of simultaneous interpretation. Moreover, ongoing work on the acquisition of expertise in interpretation, which is a highly demanding linguistic task involving rapid language switching and handling multiple simultaneous linguistic streams, will shed further light on the executive control of language in the multilingual brain.

## Author Contributions

SH realized the experience and wrote the article. AVD oversaw the experience and co-wrote the article. The two authors participated in the revision of the article for publication.

## Conflict of Interest Statement

The authors declare that the research was conducted in the absence of any commercial or financial relationships that could be construed as a potential conflict of interest.

## Footnotes

1 The results of the analysis by replacing the age variable by the year of experience variable are similar. Indeed, the age and the number of years of experience are very much correlated (r = 0.952).
